# Hierarchical Network Connectivity and Partitioning for Reconfigurable Large-Scale Neuromorphic Systems

**DOI:** 10.3389/fnins.2021.797654

**Published:** 2022-01-31

**Authors:** Nishant Mysore, Gopabandhu Hota, Stephen R. Deiss, Bruno U. Pedroni, Gert Cauwenberghs

**Affiliations:** ^1^Integrated Systems Neuroengineering Laboratory, Department of Bioengineering, University of California, San Diego, La Jolla, CA, United States; ^2^Department of Electrical and Computer Engineering, University of California, San Diego, La Jolla, CA, United States; ^3^Institute for Neural Computation, University of California, San Diego, La Jolla, CA, United States

**Keywords:** distributed processing, neuro-inspired computing, brain-scale networks, hierarchical connectivity, network compiler for neuromorphic systems, compute-balanced partitioning, hardware-aware partitioning

## Abstract

We present an efficient and scalable partitioning method for mapping large-scale neural network models with locally dense and globally sparse connectivity onto reconfigurable neuromorphic hardware. Scalability in computational efficiency, i.e., amount of time spent in actual computation, remains a huge challenge in very large networks. Most partitioning algorithms also struggle to address the scalability in network workloads in finding a globally optimal partition and efficiently mapping onto hardware. As communication is regarded as the most energy and time-consuming part of such distributed processing, the partitioning framework is optimized for compute-balanced, memory-efficient parallel processing targeting low-latency execution and dense synaptic storage, with minimal routing across various compute cores. We demonstrate highly scalable and efficient partitioning for connectivity-aware and hierarchical address-event routing resource-optimized mapping, significantly reducing the total communication volume recursively when compared to random balanced assignment. We showcase our results working on synthetic networks with varying degrees of sparsity factor and fan-out, small-world networks, feed-forward networks, and a hemibrain connectome reconstruction of the fruit-fly brain. The combination of our method and practical results suggest a promising path toward extending to very large-scale networks and scalable hardware-aware partitioning.

## 1. Introduction

There has been a growing interest in the scientific community to attain a comprehensive understanding of the brain (Markram et al., 2011; Kandel et al., 2013) using actual *in-vivo* brain recordings or simulation models using spiking neural networks (SNNs). However, simulating such large brain-size networks (Ananthanarayanan and Modha, 2007) with massive size and complexity of neurons and interconnections between them is extremely challenging to realize using the computational capability of today's digital multiprocessors. Thus, extreme-scale distributed computing is being explored as an alternative route to the physical limitations in traditional computing methods.

Computing systems with high-bandwidth interconnects between individual compute elements are crucial for such enormously distributed processing, and to demonstrate performance efficiency at brain scale. Such processing architectures are also energy-efficient edge ML acceleration tasks such as audio, image, and video processing. Data movement through a Network-On-Chip (NoC) becomes the most challenging part in the synchronization and event exchange of many-core spiking processors. This communication becomes the limiting factor in the processing, while the computation scales linearly with the number of cores (Musoles et al., 2019). To minimize the inter-core communication, we require both hardware optimization as well as an efficient compiler to generate an optimal network partitioning and mapping to the available computational resources. Distributed computing for spiking networks is the most efficient when performed with a combination of load-balancing, with which computation at the lowest latency is realized for any given network; and inter-core connectivity minimization, which ensures the most optimum reduction in traffic volume over the network.

We use an extended version of the hierarchical address-event routing (HiAER) architecture (Park et al., 2017) for scalable communication of neural and synaptic spike events between different cores. HiAER implements a tree-based interconnect architecture of fractal structure in the connectivity hierarchy, where the communication bandwidth at each node is relatively constant at each level in the hierarchy due to decreasing fan-out at increasing levels. The notation *L*_*i*_ corresponds to the *i*'th level of communication hierarchy. The lowest level (*L*_0_) in this hierarchy represents the intra-core communication, which is just governed by a synaptic routing table (postsynaptic neuron destinations stored in the local memory allocated to the same core), which incurs no routing cost. As we organize these cores within the larger system, we can create further hierarchy, i.e., *L*_1_ (communication within a cluster of cores) and *L*_2_ (communication between clusters). For example, in order to arrange 32 cores onto two layers of hierarchy, we can arrange them into eight *L*_2_ clusters of four *L*_1_ cores, 16 *L*_2_ clusters of two *L*_1_ cores, or any other arrangement of two factors with a product of 32. This type of organization allows us to easily map our cores and routing nodes to hardware, where there are specific routing requirements and network connectivity while obviating the need for all-to-all connections through an extremely high bandwidth interconnect. We can extend our hierarchically structured communication network further by adding additional levels of hierarchy.

Peak efficiency in near-memory or in-memory compute architectures requires all compute cores to get efficiently utilized, assuming a network structure that is locally dense and globally sparse. The previously discussed HiAER protocol for routing of neural spike events allows network connectivity that scales to networks of virtually unlimited size, due to decreasing connectivity density with an increasing distance that permits near-constant bandwidth requirements in event routing across the spatial hierarchy. An ideal neuromorphic computing system attains both the computational efficiency of intra-core dense local connectivity with near-memory compute cores, and the functional flexibility of HiAER sparse long-range connectivity. An open problem in the practical realization of this system is to efficiently map a given network with arbitrary topology onto the implemented hierarchy of cores with rigid dimensions. This requires automated means to partition the network in such a way to maximally align its connectivity with maximal fill density of the connectivity matrices within cores, and minimal communication of neural events across cores.

**Figure 1:**
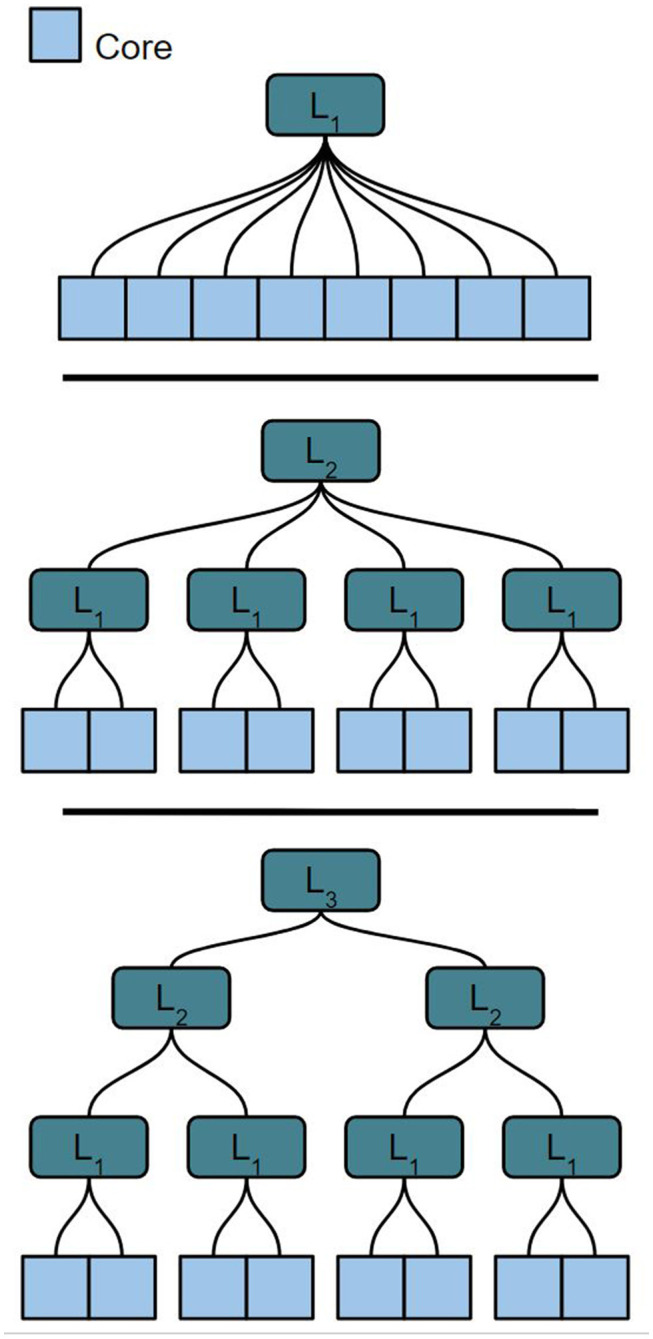
Different potential tree-based HiAER network configurations for 8 cores with varying *L*_*i*_ and a fixed core count *K*.

The optimal partitioning method to align large, arbitrarily structured networks onto a scalable HiAER topology must satisfy several conditions. First, the partitioning scheme should be fast and scalable to different levels of the HiAER communication scheme. A network should be able to be partitioned over *K* cores. Neurons in these cores will use different levels of off-core communication depending on the location of the destination core in the communication hierarchy. We assume that each neuron has equal processing time in the cores. The total size of the network can be written as *n*_0_*K, with *n*_0_ being the number of neurons in each of the *K* cores. Depending on hardware constraints and space that the user provides, the partitioning method should be able to partition over different possible values of *n*_0_, *K*, and *L*_*i*_. Figure 1 shows different potential configurations of the network around *K* cores. The partitioning method should be able to find an optimal partition and core configuration for each case shown.

Additionally, the network of *N* neurons distributed across *K* cores must produce balanced partitions where each core contains roughly *N*/*K* neurons. Cores with too many neurons will be bottlenecks in network performance due to longer processing time, while cores with too few neurons will be underutilized. Distributing the neurons in a balanced fashion allows for balanced processing time among the cores and maximizes the overall network speed.

Finally, the partitioning method should result in savings in both memory and communication across cores. This means that the neurons should be arranged in a way such that cross-core communication is minimized, which involves both grouping neurons with similar incoming connections inside the same core, as well as minimizing cross-core communication. These savings must be applicable to partitions that are at single or multiple levels of hierarchy. In most partitioning methods, minimum edge-cut is used as the metric for judging the quality of the partition and can be defined as the number of edges whose incident vertices belong to different partitions. However, minimum edge-cut does not suffice to describe the network communication when using AER or mask bits due to the fact that multiple connections can be encoded in the same communication packet. For our evaluation, we do not use the minimum edge-cut in order to evaluate the quality of the partition, and instead, we use our own set of routing rules defined for the HiAER network routing.

For a given input network, it is difficult to partition based on the activity of each neuron in the network. Depending on the neuron model, inputs, and synaptic strengths, networks of the same underlying structure can behave unpredictably. Because of this, algorithms like SNEAP (Li et al., 2020) use spike traces from simulations of the network in order to better partition based on the network activity. In this work, we chose to develop a partitioning strategy that only depends on the connectivity of the input graph. For this reason, it makes sense that a partitioning method for reconfigurable hardware should be performed on a purely topological basis. Various types of SNN toplogies exist, including liquid state machines (Maass, 2011), deep spiking networks (Sengupta et al., 2019), and large-scale brain models (Potjans and Diesmann, 2012). This is due to the fact that it is not feasible to simulate large-scale models for activity data every time the model needs to be partitioned. Splitting an input network into a balanced set of partitions is known as the NP-hard *balanced graph partitioning problem*. Several balanced graph partitioning approximation methods exist, including METIS (Karypis and Kumar, 1999), a multilevel partitioning scheme, which is commonly used for its speed, flexibility, and performance. Solutions like Spinner (Martella et al., 2017), which runs on Giraph (a large-scale graph analytics platform) easily scale to massive graphs and a large number of compute cores. Streaming graph algorithms such as FENNEL (Tsourakakis et al., 2014) also offer very fast balanced partitioning solutions, where vertices are partitioned one-by-one, minimizing the computation required. These algorithms run on both weighted and unweighted undirected graphs, and have typically been used to partition very large graphs of social media networks or in very large-scale integration (VLSI) circuit partitioning (Alpert et al., 1996).

There are some previous works on SNN mapping methods to neuromorphic platforms. Some of these methods include PACMAN (Galluppi et al., 2012), SCO (Lee et al., 2019), and SpiNeMap (Balaji et al., 2020). PACMAN, which is the partitioning and configuration framework for SpiNNaker (Painkras et al., 2013), partitions on a population level, and then sequentially maps the result to a huge number of ARM processors emulating SNN cores. This works well for the torus interconnect in SpiNNaker but clearly doesn't suit our hierarchical tree-based interconnects. The hierarchical tree-based interconnects are more scalable due to the fact that new branches can be added to the tree that maintains constant local bandwidth, in contrast to linear network-on-chip where congestion can arise from a large number of connections (Park et al., 2017). SCO minimizes the hardware resources for network execution but doesn't have any performance gains in reducing global communication traffic. SpiNeMap reduces the power consumption and latency for crossbar-based neuromorphic cores where the communication fabric is a single shared time-multiplexed interconnect. Previously, METIS has been used to partition spiking networks in Barchi et al. (2018b) and Li et al. (2020). However, in the former, the cortical microcircuit network used for analysis is quite small, scaled down to only roughly 4,000 neurons and seven hundred thousand synapses. In the latter, spike trace information is used as weights during the METIS partitioning, the mapping strategy used does not take into account a HiAER network structure, and the networks used are relatively small. Additionally, there is no experimentation on different toplogies of spiking neural networks. Other works, such as Barchi et al. (2018a), use several other graph partitioning methods such as spectral analysis, and simulated annealing in order to partition their input SNN, but do not extend its methods to different layers of network hierarchy. These previous works are not fully optimized for a HiAER network structure and to our knowledge, there is no existing work that has optimized the partitioning and mapping algorithm to be implemented using a HiAER framework. As HiAER offers the maximum flexibility in network connectivity as well as provides the highest scalability, our hardware-aware partitioning algorithm has the potential to scale to networks at the scale of the human brain.

## 2. Methods

In this section, we describe the hierarchical routing methods in which we use to evaluate the quality of the partitioning algorithm, as well as the hierarchical partitioning algorithm that we use for the optimal mapping of neurons to cores, and of cores to locations in the network hierarchy.

### 2.1. Routing and Network Evaluation

Our communication is based on Address-Event Routing (AER) from a presynaptic neuron in an origin core to a postsynaptic neuron in a destination core (Mahowald, 1994; Boahen, 1999). In the AER protocol, neurons communicate asynchronously whenever they spike, with weights stored locally at each postsynaptic destination. Each spike is represented by a destination address and an event time. When the destination core(s) receive this event, it is subsequently processed and the correct weights are accessed to update the postsynaptic neuron(s) in the core or to propagate further messages off-core. This configuration allows for minimum traffic because only a single connection is required per neuron to each of its destination cores, regardless of the postsynaptic fan-out at each destination core. From the router's perspective, communicating with one neuron in the destination core is equivalent to communicating with all neurons in the destination core. This is a key factor in our partitioning evaluation methods.

**Figure 2:**
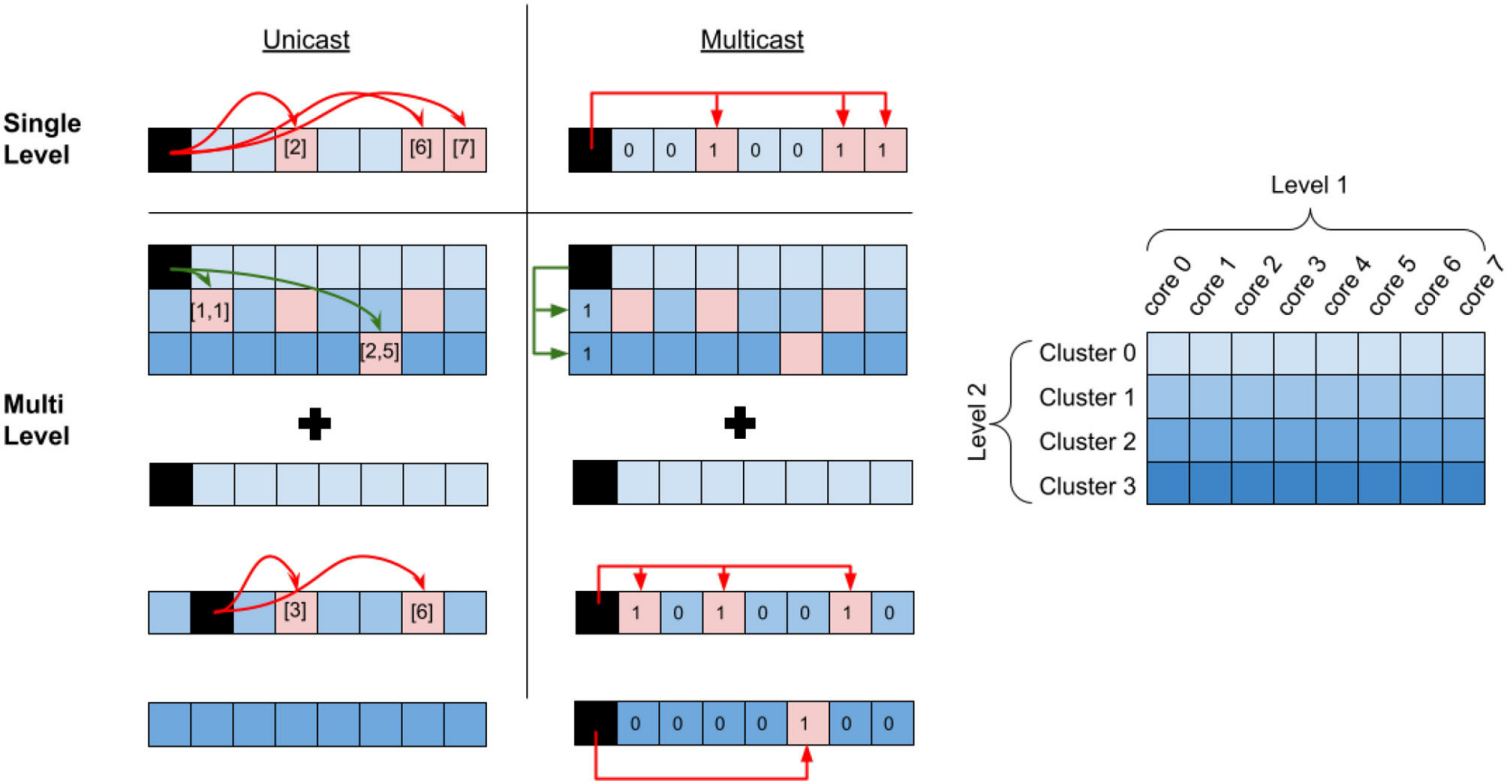
Unicast vs. Multicast communication. The red arrows describe communication within a cluster (*L*_1_ communication) and the green arrows describe communication between the clusters (*L*_2_ communication). In the unicast example, each postsynaptic destination core address is explicitly defined in the communication packet (e.g., [1,1] and [2,5]), and each crossing of hierarchy requires an explicit message. In the multicast example, only the destination mask is defined in the communication packet. In both multi-level cases, “relay” connections are needed in order to route to the destination core, which routes to the correct core at the local level of hierarchy. In the unicast case, a single relay connection is needed over *L*_2_ to core [1,1] and two relay connections are required in this local *L*_1_ to route to cores [3] and [6]. In the multicast case, a single relay connection is needed over *L*_2_ and additionally over each *L*_1_.

Figure 2 shows the system organization with cores communicating through *L*_1_ and *L*_2_ interconnects. The communication similarly extends into additional levels of hierarchy. Due to potentially different physical modes of communication at different levels of the system, we assume that communication cost increases with the level of hierarchy. Our hierarchical partitioning method is flexible in that we can choose the number of cores and choose the placement of the cores in the different levels of the communication hierarchy.

We define two alternate methods of communication for our evaluation. The first method, which we call *multicast* communication uses mask bits at each level of hierarchy in order to determine which destination cores to communicate with. The cores in the same level of hierarchy have a shared address in order to reduce the routing complexity and cost. This means that a presynaptic neuron in a source core connected to multiple destination cores requires only a single message, provided that those cores are all connected. Figure 2 shows a single message used to communicate with every core in the single-level multicast. Communicating to core(s) in a different *L*_1_ requires an intermediate connection over *L*_2_ to the same core index. In Figure 2, this is shown in the multilevel multicast. From here, an additional intermediate connection at each destination *L*_1_ will route to the correct destination cores (this connection is not required if all destination cores can be reached with the *L*_2_ message). We call these intermediate connections “relay” connections. This approach scales up to the highest level of communication. With *i* levels of hierarchy, the worst-case communication will require *i* relay connections. The advantage of this approach is that it constrains the communication within and between levels to drastically improve the speed of message processing. Additionally, using the mask bits allows for a reduction in the overall number of messages over the network.

The second method, which we call *unicast* is where cores have the ability to connect to any other core in the network. Each unique crossing of a hierarchy requires its own unique message, unlike the multicast case where a single message was needed. For multilevel communication, relay connections are still needed to route to the correct destination core. This is shown in the multilevel Figure 2 example, where crossing the *L*_2_ hierarchy is only done once for each cluster, with a relay message to core [1,1], and additional relays to cores [3] and [6] in the local cluster. The message to [2,5] is not considered a relay connection, as there is no further local fan-out. In unicast communication, significantly more relay connections may be needed in order to correctly fan-out to all destination cores. While unicast messaging doesn't constrain communication the router must be able to handle a high volume of these messages within a reasonable time, which might be difficult. Additionally, each message packet needs to dedicate space to contain the correct destination core address.

We evaluate the quality of the partitions using both communication methods to calculate the number of messages in each level of hierarchy in order to find the total cost for the partition. Since this system organization and routing is unique, we compare all experiments to balanced random assignment, where ≈*N*/*K* neurons are chosen at random and assigned to each core, and quantify the quality of the partitioning by how many messages the algorithm can beat the balanced random assignment at each level of hierarchy.

### 2.2. Hierarchical Partitioning

The partitioning method introduced here is based on METIS (Karypis and Kumar, 1999), although in principle it can work with any balanced graph partitioning algorithm. METIS is a multi-level graph partitioning scheme that uses either multilevel recursive bisection or multilevel *k*-way partitioning algorithms. METIS works in three stages. The coarsening stage transforms the initial graph *G*_0_ into sequentially smaller graphs *G*_*k*_. In the next step, *G*_*k*_ is then quickly partitioned. Finally, the refinement stage projects the partition back to each level, with greedy refinement at each step. We chose METIS specifically due to its ease of use, speed, and performance. We restrict our use of METIS to *k*-way partitioning in order to compute a *k*-way partitioning such that the edge-cut of the graph is minimized (even though this is not our metric for analysis). The overall communication volume is minimized by running METIS on the network connectivity graph. This is a generic step that can reduce the number of edges for targeted graph partitions, and produces our baseline partition. This requires no simulation of the network and is purely based on the unweighted and undirected connectivity of the input. Beyond this reduction, our hierarchical METIS partitioning method obtains further reduction of communication volume, which suits our optimization goal for the HiAER routing scheme.

**Figure 3:**
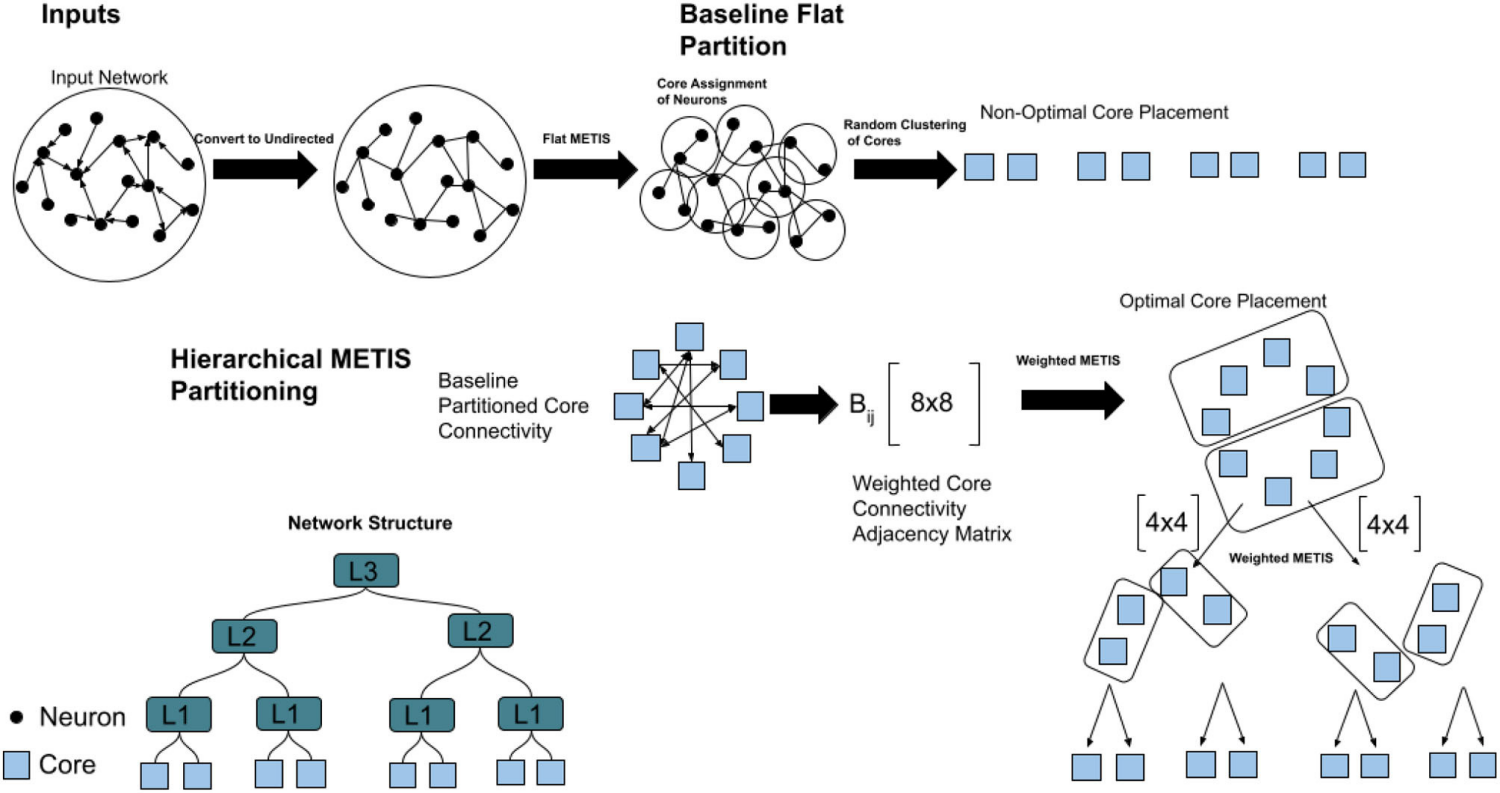
Hierarchical network partitioning algorithm and baseline flat partitioning algorithm.

Figure 3 shows the procedure for hierarchical partitioning. We first feed the un-directed input network and the number of partitions required to the METIS algorithm. This gives balanced partitions and class labels for each of the n neurons in the network. Each neuron is assigned to its class label, which designates the core it belongs to. We compute the “flat” partitioning by randomly mapping cores to locations in the hierarchy of partitions. We can evaluate our network with this baseline, which would be the optimal partitioning for a router with a single layer of hierarchy. However, randomly mapping cores to locations with multiple levels of hierarchy is not optimal, and can lead to a significant burden on the router. Instead, we define an algorithm for hierarchical partitioning onto the multiple layers of hierarchy. We compute the number of unique connections for each neuron in a core to a postsynaptic destination outside of the core. This is then compiled in an adjacency matrix *A*_*ij*_ which dictates the number of these connections from core *i* to core *j*. This adjacency matrix holds the directed cross-core communication of the entire network. In order to create an undirected adjacency matrix, we create a new adjacency matrix *B*_*ij*_ where each element is the sum of *A*_*ij*_ and *A*_*ji*_. This is a symmetric matrix that highlights the total cost of communication between each core.

We then iterate through each level of the hierarchy. We run a weighted version of METIS on this adjacency matrix, where the weight is the cost of communication between 2 cores as computed earlier. We select the number of elements we want to partition at the top-most level of the *i*′th level of hierarchy and compute the assignments. For each partition, we take the *h* nodes that were assigned to that partition, and group their entries into a separate adjacency matrix, removing all connections to cores that do not have the same assignment. This weighted matrix is then partitioned with METIS as before into *i*−1 levels of hierarchy, and this process is repeated until the lowest level of hierarchy has been reached.

The initial flat partitioning algorithm allows for neurons to be mapped to cores in the network. The motivation for the hierarchical partition is that the flat partitioning itself is not enough aware of the network structure, and thus will have poor results for minimizing traffic across cores. The hierarchical step allows for cores to be mapped to slots in the hierarchies, which allows for the best possible arrangement of cores in the network. The top-down approach for partitioning ensures us that we are minimizing traffic from the highest level down, due to our assumption that the higher levels of communication are the most costly. Throughout the paper, we will refer to hierarchical partitioning using AxBxC notation, where the cores are first partitioned into groups of size A, then of size B, and finally of size C. In this paper, we constrain the partitioning of the network to up to three levels, even though the methods we present could potentially partition the network on a network architecture with a deeper hierarchical structure.

### 2.3. Synthetic Network Generation

**Figure 4:**
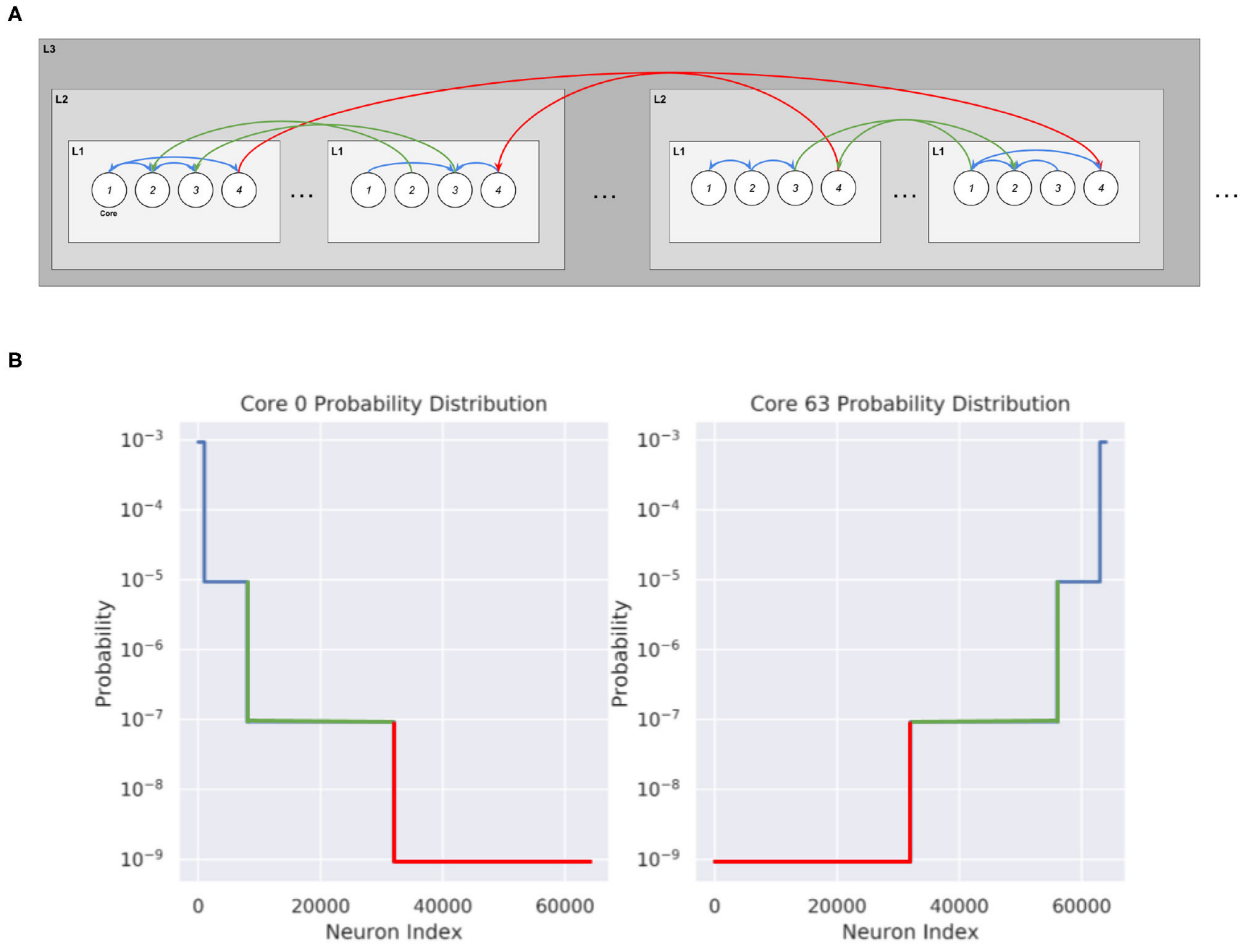
Synthetic network connectivity and probability distribution of cores when λ = 0.01. **(A)** Hierarchical Routing structure within the cores obtained from the Synthetic Network Partitioning. The probability of connection decreases as the level of hierarchy increases. **(B)** Probability distribution of 2 cores in a 64,000 neuron Synthetic network, organized into 2×8×4. Each core contains 1,000 neurons, with core 0 containing the first 1,000 neurons of the network and core 63 containing the last 1,000 neurons of the network.

We create various synthetic networks in order to test the partitioning algorithm. These networks consist of cores with local densely connected neurons inside each core and can be generated for different levels of hierarchy. We first generate 1,000 neurons in each of the *K* cores that we want to create. To connect these networks, we draw from a probability distribution where *u* is the normalizing constant, λ indicates the spread factor, and *n*_*i*_ gives the number of possible postsynaptic destinations at the *i*'th level of hierarchy. This is shown in Figure 4:


(1) $$\begin{array}{rcl} u\left(n_{0}+n_{2}\lambda +n_{2}\lambda^{2}+n_{3}\lambda^{3}\ldots \right) & = & 1 \end{array}$$

(2) $$\begin{array}{rcl} u\lambda^{i}n_{i} & = & p\left(n_{i}\right) \end{array}$$

At the lowest level of hierarchy, *n*_0_ = 1,000, which indicates local connections. Then, we designate how many neurons are at each level of hierarchy before solving and normalizing in order to create a probability distribution for each neuron in the core. The total probability of connecting to any neurons in a certain level of hierarchy is given by the normalizing factor multiplied by the spread factor at that level of hierarchy and by the total number of neurons at that level of hierarchy.

The spread factor λ indicates the overall spread of the network. With λ≪1, the PDF of each core will strongly favor local connections with very few connections to neurons at higher levels of hierarchy. At the opposite end, a λ = 1 indicates a completely randomly connected graph, where each neuron is equally as likely to connect to any other neuron in the network. Partitioning on a randomly connected graph should not be expected to give significant improvements in message reduction. On the other hand, the partitioning should be able to exploit the topology of networks with smaller lambda, and significantly reduce communication. These synthetic networks are useful to create because the ground truth for the ideal partitioning is known, which enables us to check the performance of the partitioning method.

## 3. Results

The hierarchical partitioning algorithm was tested with a wide variety of networks in order to show invariance to input topological structure. All partitioning results displayed are the mean of 5 trials with the METIS algorithm on the same graph. The standard deviations obtained were typically very small (≪1%) and thus have been not been listed. The experiments were done on a computer with an Intel I7-8700 processor and 64 GB DDR4 SDRAM.

### 3.1. Partitioning on Synthetic Networks

We generate and produce results for various synthetic networks. In each synthetic network, there are 1,000 neurons in each node. We vary the average fan-out for each neuron to 64, 128, and 256 postsynaptic destinations. The postsynaptic destinations are randomly sampled from a unique probability density function for the neurons in the presynaptic core. Once the connections are completed, the network neuron indexes are randomized and sent to the partitioning algorithm for partitioning and evaluation. We consider synthetic networks created with 2 and 3 levels of hierarchy. For the network with 2 levels of hierarchy, we generate a network with 4 *L*_2_-groups of 8 *L*_1_-nodes each, which has a total size of 32,000 neurons and n maximum average of about 8,192,000 synapses. For the network with 3 levels of hierarchy, we generate a network with 2 *L*_3_-groups, each containing 4 *L*_2_-groups, which further each contain 8 *L*_1_-cores each, and a network with 8 *L*_3_-groups, each containing 4 *L*_2_-groups with 8 *L*_1_-cores each. These networks have a size of 64,000 and 256,000 neurons and a maximum average of 16,384,000 and 65,536,000 synapses, respectively. This is an extended and revised version of a preliminary conference report that was presented (Mysore et al., 2021), which was constrained to 2 layer partitioning only focused on multicast communication.

**Figure 5:**
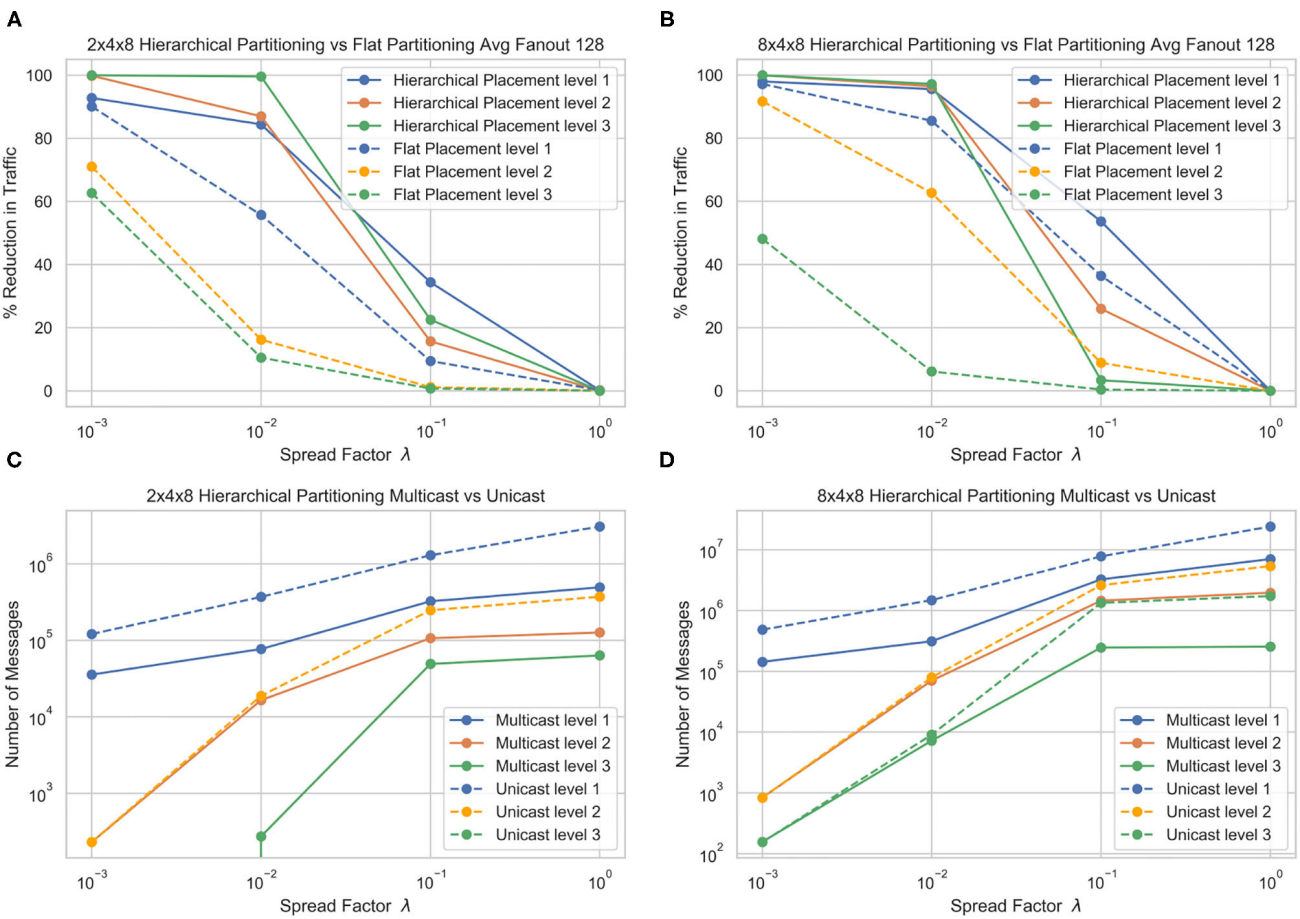
Traffic reduction plots for different hierarchy structures and comparison of cost under two different evaluation methods, for flat core placement **(A)** and hierarchical **(B)** core placement. The number of messages generated by multicast **(C)** and **(D)** unicast messaging in each layer are also shown.

Figure 5 shows the performance of the hierarchical partitioning on the 3 layer partitioning. At each level of sparsity except λ = 1, the hierarchical partitioning method beats the flat baseline partitioning. Partitioning into a bigger *L*_3_ seems to incur a faster dropoff in messages as the spread factor increases. As expected, at a spread factor of 1, the partitioning methods converge to random, and there is no improvement over random partitioning of the network. At a low spread factor, the algorithm is able to almost perfectly partition the network, reducing almost all the *L*_3_ communication and significantly reducing the *L*_2_ and *L*_1_ communication.

Figure 5 also shows the number of messages generated by the multicast and unicast routing schemes. In the 2 × 4 × 8 partition, there is a significant reduction of messages in the *L*_1_ and *L*_2_ messaging, but the *L*_3_ stays the same. This is because the *L*_3_ boundary is always crossed, and since there is only 1 other destination, the evaluation methods are equivalent. This can be observed in the 8 × 4 × 8 partition, where the *L*_3_ messages are significantly higher in unicast than multicast since there are 7 additional destinations. We see that multicast messaging allows for a greater reduction in overall messages, although the hierarchical partitioning significantly reduces the number of messages in both evaluation methods.

### 3.2. Small-World Networks

While the synthetic networks offer an excellent baseline to evaluate the efficacy of the partitioning method, it is essential to test on other networks that are not generated with biases toward an expected hierarchical structure. To this extent, we generate various small-world networks for the evaluation of the partitioning algorithm. Small-world networks are a common topological basis for modeling anatomical connections in the brain and thus are common networks to model spiking neural network connectivity (Bassett and Bullmore, 2007). Starting with *n* nodes, each node in the ring is connected to *k* of its nearest neighbors. Each edge is then replaced with a probability *p* with a new edge which is uniformly sampled from the collection of neurons. Based on the parameters, the rewiring algorithm creates a network that is neither regular nor random. For our analysis, we vary *n* and *k* in order to show the efficacy of the partitioning on various small-world graphs. We show the performance of the hierarchical partitioning algorithm with various configurations in order to show that it is beneficial with any network hierarchy.

**Figure 6:**
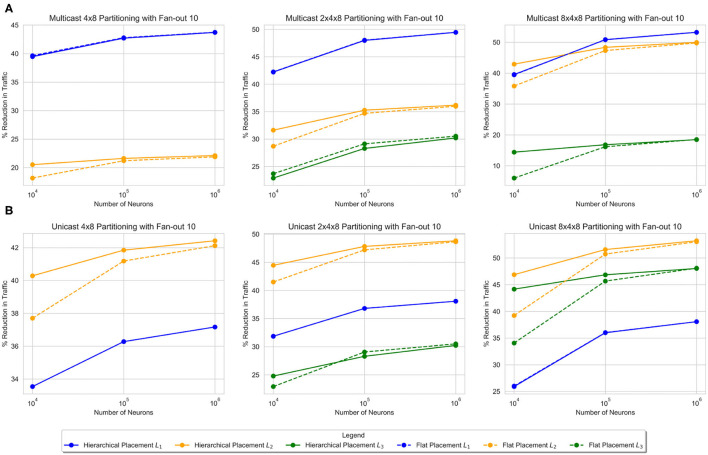
Traffic reduction plots for small-world networks with different hierarchy structures and comparison of cost under multicast **(A)** and unicast **(B)** evaluation methods.

Figure 6 shows the results from small-world networks with a fan-out of 10. Three networks are tested with up to 1 million neurons. In most cases, the hierarchical partitioning method beats the flat partitioning method. As the number of neurons increases, both the flat and hierarchical partitioning methods seem to converge to the same values. Additionally, as the number of neurons increases, the performance of the increases at each level and for each partitioning layout. While both unicast and multicast provide a reduction in message volume, multicast communication has significantly less message traffic. Table 1 shows similar results for a small-world network with a fan-out of 256. The results are significantly less impressive, primarily because when an edge is rewired, it chooses a random destination in the network. A fan-out of 256 is enough to incur significant randomness to the graph and reduce the partitioning quality. Nevertheless, there is still an observable reduction in unicast communication, primarily at the level 1 hierarchy. This is due to the fact that the hierarchical partitioning algorithm is able to significantly reduce the number of relay connections.

**Table 1:** Small-world network hierarchical partitioning results (fan-out = 256).

**Synthetic network**	**Hierarchy level**	**Percent message reduction (Multicast)**			**Percent message reduction (Unicast)**		
		**N = 10^**4**^**	**N = 10^**5**^**	**N = 10^**6**^**	**N = 10^**4**^**	**N = 10^**5**^**	**N = 10^**6**^**
**4 × 8**	Level 1 Level 2	6.58 0.02	5.38 0.75	3.82 0.92	42.30 4.95	27.64 4.56	21.61 3.50
**2 × 4 × 8**	Level 1 Level 2 Level 3	16.23 2.87 0.92	13.55 1.56 0.33	7.19 1.94 1.21	44.22 11.19 0.99	39.44 10.12 0.26	32.14 6.53 0.26
**8 × 4 × 8**	Level 1 Level 2 Level 3	14.73 2.83 0.90	12.88 1.49 0.25	7.35 2.20 1.28	44.20 14.40 0.96	39.43 12.25 6.54	32.13 0.31 1.25

### 3.3. Deep Feedforward Network

**Figure 7:**
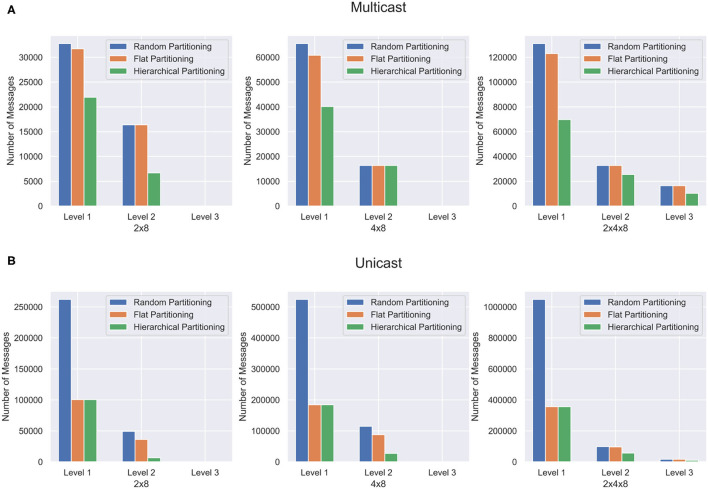
Partitioning results for the deep feedforward network. The network is partitioned in three different ways and communication cost reduction is demonstrated using **(A)** multicast and **(B)** unicast.

Deep feedforward networks are commonly used today for various machine learning tasks. These networks typically consist of stacked perceptron layers that feed into each other until they reach an output layer, where a probabilistic decision is made. The topology is also commonly used for various deep spiking neural networks such as in Wu et al. (2020). The hierarchical partitioning algorithm is run on a sample deep feedforward network. The network contains 8 fully connected layers, each with 2,048 neurons, and a single output layer of 1,000 neurons. These networks contain no feedback connections. Figure 7 shows the results of the deep feedforward network when with different partitioning.

One interesting observation is in the *L*_1_ messages. In the unicast example, the hierarchical partitioning method barely performs better than flat partitioning in each case. However, the multicast offers a significant reduction in *L*_1_ communication volume. This is because hierarchical partitioning can significantly reduce the number of relay connections needed when it places cores since the communication is masked, while the unicast cannot take advantage of this.

### 3.4. Fly Hemibrain Connectome

**Figure 8:**
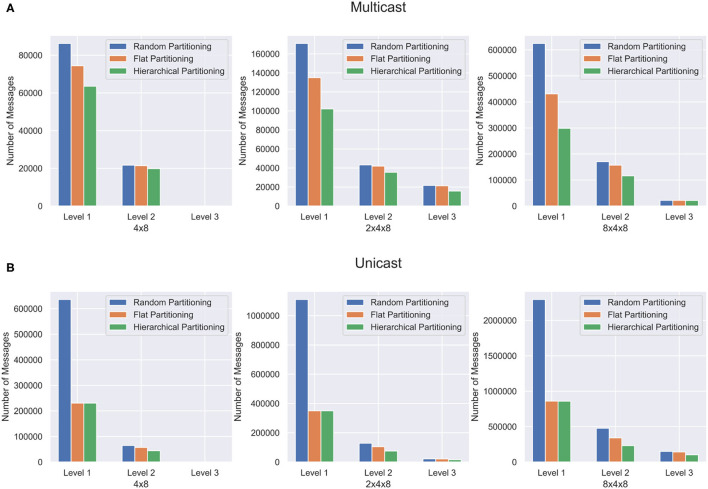
Partitioning results for the fly hemibrain network. The network is partitioned in three different ways, and communication cost reduction is demonstrated using **(A)** multicast and **(B)** unicast.

Synthetic Networks are excellent for validating the partitioning method but are not representative of the structures found in a real brain. In order to capture the effectiveness of the hierarchical partitioning method on real neuronal networks, we use the fly hemibrain connectome (Xu et al., 2020). The connectome reconstruction dataset contains about twenty-thousand neurons as well as over three million synapses, obtained through a combination of Electron microscopy, segmentation pipeline, and novel synapse prediction methods. The dataset contains thousands of cell types spanning over several regions in the brain. This representation depicts a realistic biological network topology for partitioning. The connectivity of the dataset was extracted and converted into a graph format. Figure 8 shows the partitioning results for the fly hemibrain. The network is partitioned into 4 × 8, 2 × 4 × 8, and 8 × 4 × 8 structures. The 4 × 8 and 2 × 4 × 8 hierarchical partition provides significantly better results than flat and random partitioning at all levels of communication. The 8 × 4 × 8 partition is able to provide a significant reduction in *L*_1_ and *L*_2_ messages but does not reduce the *L*_3_ communication as much using multicast. This could be because with only twenty-thousand neurons, split across 256 cores, the partitioning is too wide and it is not possible to reduce the amount of communication in this level of hierarchy.

### 3.5. Partitioning Time

**Figure 9:**
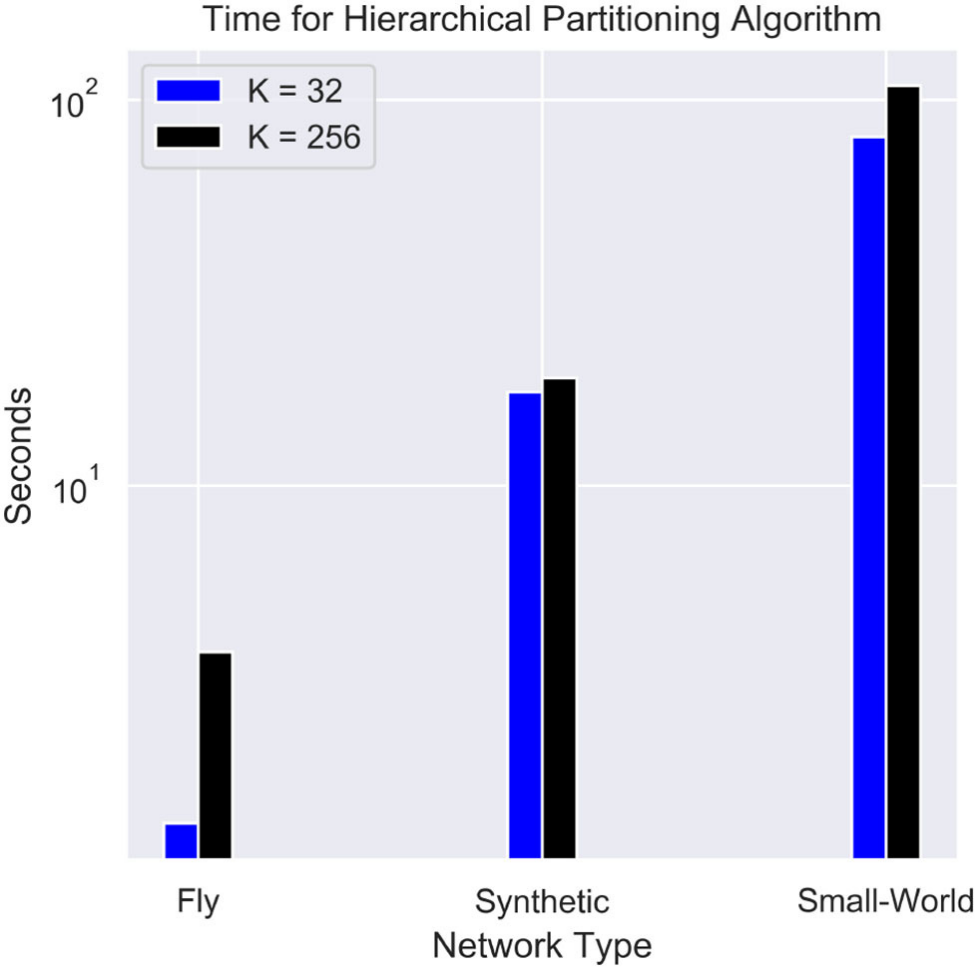
Timing for the hierarchical partitioning of each network. The synthetic network used contains 256,000 neurons and 65,536,000 synapses, and the small-world network contains 1 million neurons and a fan-out of 256.

The time complexity for the partitioning is often an important parameter when implementing real-time workload. Figure 9 shows the performance of the hierarchical partitioning algorithm. The majority of the time is taken by the baseline METIS algorithm. The hierarchical partitioning of the cores takes on the order of 0.1 s since the METIS partitioning is only done on a *K* × *K* adjacency matrix. Partitioning into fewer cores results in a significant improvement in speed. Other balanced partitioning algorithms such as parMETIS (Karypis et al., 1997) and FENNEL (Tsourakakis et al., 2014) can be used as alternatives to speed up partitioning, but either require more compute nodes or can potentially worsen the quality of the partitioning.

## 4. Discussion

The goal of this work was to create and evaluate a flexible method of hierarchical partitioning for use in large-scale neuromorphic systems. We designed and developed an algorithm for performing this partitioning on various networks including synthetically generated networks, small-world networks, deep feedforward networks, and the fly hemibrain. In all cases, we observed a significant improvement of the hierarchical partitioning method over randomly balanced neuron placement and even flat METIS partitioning. The method is very flexible and scales to any number of layers of hierarchy, allowing fast reconfigurability and improved performance for neuromorphic systems.

The hierarchical partitioning algorithm mainly takes advantage of the structure of an input network. In many cases, graphs that have a large number of random connections will not result in a reduction in message traffic. This was observed both in the synthetic networks at high spread factor and in the small-world graphs with large fan-out. However, as demonstrated in the fly hemibrain connectome, real networks can be partitioned and simulated with significant benefits. Further analysis must be done in order to validate the advantages of this partitioning method on data from more complex organisms, such as mice and humans.

We also evaluated the cost/benefit of unicast and multicast communication protocols over the network. While unicast message reduction often benefits significantly more from hierarchical partitioning than from multicast, overall multicast communication allows for a drastic reduction in the overall number of messages and total network traffic. Multicast communication also benefits from requiring a fixed mask in each message, while unicast communication requires the full destination address. With the optimal placement generated from the hierarchical partitioning algorithm, multicast communication typically can take better advantage of the structure to reduce the number of relay messages.

Many neuromorphic systems such as Loihi (Davies et al., 2018) and TrueNorth (Akopyan et al., 2015) use a mesh NoC on a single chip. These topologies are popular because of their low complexity and planar 2D layout properties. It is easier to generate the most optimal placement of the neurons and cores in this type of mesh. However, large planar systems may suffer from excessive hop counts when communicating end-to-end. The hierarchical structure we discussed has the advantage of improving bandwidth for long-distance connections. For each level of hierarchy, the communication sparsity and address-space both increase in each level, multiplying to a constant bandwidth at each level of hierarchy. Assuming constant average fan-in, fan-out, and event-rate for each neuron, the hierarchical connectivity that we presented will scale linearly with the number of neurons in the network. If additional cores are needed for larger networks, the partitioning method has proven to be able to find an optimal arrangement for a particular hierarchical structure of cores. However, this structure may be significantly worse than the most optimal hierarchical structure for the network. Finding the optimal hierarchical structure may require prior information about the spiking network connectivity.

One area of further study is the idea of an optimal hierarchical structure for the input network. If *N* neurons can be placed inside of a single core, then placing all *N* neurons in a single core eliminates network traffic but takes longer simulation time. On the other hand, distributing the neurons over all available cores will improve the speed of simulation to the extent that the router can handle all of the messages. In order to further validate the partitioning method, we plan to test this on reconfigurable neuromorphic digital hardware such as a field-programmable gate array (FPGA) such as in Pedroni et al. (2020), and analyze the total speedup and communication volume over the router, as well as try to identify quantitative guidelines to determine the optimal partition. Additionally, further evaluation can be done on more complex network topologies, such as spiking Convolutional Neural Networks (Lee et al., 2018), and larger real biological networks. This opens the door to fast and efficient neural simulations with neuromorphic hardware on a very large scale.

## Data Availability Statement

The raw data supporting the conclusions of this article will be made available by the authors, without undue reservation.

## Author Contributions

NM implemented the algorithm, created and collected data, and collected results. All authors wrote and reviewed the manuscript.

## Funding

This research was supported by the National Science Foundation, Office of Naval Research, Western Digital Corporation, and Defense Advanced Research Projects Agency.

## Conflict of Interest

This study received funding from Western Digital Corporation. The funder was not involved in the study design, collection, analysis, interpretation of data, the writing of this article or the decision to submit it for publication. All authors declare no other competing interests.

## Publisher's Note

All claims expressed in this article are solely those of the authors and do not necessarily represent those of their affiliated organizations, or those of the publisher, the editors and the reviewers. Any product that may be evaluated in this article, or claim that may be made by its manufacturer, is not guaranteed or endorsed by the publisher.
